# Endocrine Treatment for Breast Cancer Patients Revisited—History, Standard of Care, and Possibilities of Improvement

**DOI:** 10.3390/cancers13225643

**Published:** 2021-11-11

**Authors:** Naiba Nabieva, Peter A. Fasching

**Affiliations:** 1 Department of Gynecology and Obstetrics, Erlangen University Hospital, Comprehensive Cancer Center Erlangen-EMN, Friedrich-Alexander University Erlangen-Nürnberg, 91054 Erlangen, Germany; naiba.nabieva@fau.de; 2 Novartis Oncology, Novartis Pharma GmbH, 90429 Nuremberg, Germany

**Keywords:** breast cancer, endocrine treatment, tamoxifen, aromatase inhibitor, fulvestrant, CDK 4/6 inhibitor, ribociclib, palbociclib, abemaciclib, PI3K inhibitor, alpelisib, mTOR inhibitor, SERD, compliance

## Abstract

**Simple Summary:**

Tamoxifen, aromatase inhibitors, and fulvestrant are the main drugs that have been used for decades in the treatment of patients with endocrine-therapy-sensitive breast cancer. Due to the findings of recent studies and the approval of novel substances for the treatment of this patient population, the established standards of endocrine therapy are changing. Considering signaling pathways such as the PI3K/AKT/mTOR or the CDK4/6 pathway, as well as resistance mechanisms and substances analyzed against these, endocrine treatment of hormone-receptor-positive breast cancer is on the brink of a new era. This review provides an overview of the history of endocrine treatment, clarifies its role in the present standard of care, and discusses the possibilities of improvement.

**Abstract:**

Purpose of review: Due to the findings of current studies and the approval of novel substances for the therapy of hormone-receptor-positive breast cancer patients, the established standards of endocrine treatment are changing. The purpose of this review is to give an overview of the history of endocrine treatment, to clarify its role in the present standard of care, and to discuss the possibilities of improvement. Recent findings: Tamoxifen, aromatase inhibitors, and fulvestrant are the main drugs that have been used for decades in the therapy of hormone-receptor-positive breast cancer patients. However, since a relevant number of women suffer at some point from disease recurrence or progression, several novel substances are being investigated to overcome resistance mechanisms by interfering with certain signaling pathways, such as the PI3K/AKT/mTOR or the CDK4/6 pathways. mTOR and CDK4/6 inhibitors were the first drugs approved for this purpose and many more are in development. Summary: Endocrine treatment is one of the best tolerable cancer therapies available. Continuous investigation serves to improve patients’ outcomes and modernize the current standard of care. Considering the resistance mechanisms and substances analyzed against these, endocrine treatment of hormone-receptor-positive breast cancer is on the brink of a new era.

## 1. Introduction

Endocrine treatment (ET) of breast cancer (BC) was one of the first implementations of an individualized treatment of cancer. At the end of the 19th century, Sir George Thomas Beatson first discovered the positive influence of a bilateral oophorectomy on the development of breast cancer lesions in women with advanced disease, and ET was born [1]. Over time, research on antihormonal substances progressed and it was discovered that only patients with an expression of hormone receptors received any benefit from therapy with tamoxifen [2]. The implementation of a test that assesses the expression of hormone receptors and enables an appropriate therapy decision [3] was, besides testing for the human epidermal growth factor receptor 2 (HER2) and a subsequent trastuzumab treatment [4], the beginning of individualized therapies for BC patients.

The efficacy of ET was first evaluated in women with advanced breast cancer (aBC). In addition to tamoxifen and the three aromatase inhibitors (AIs) anastrozole, letrozole, and exemestane, with fulvestrant, a selective estrogen receptor degrader (SERD), there are several ET options available for aBC patients [5,6,7]. While in a metastatic treatment setting the primary objective is to improve progression-free survival (PFS) and overall survival (OS), while maintaining quality of life (QoL) at the same time, the goal of adjuvant ET is to reduce the disease recurrence and mortality rates of hormone-receptor-positive early breast cancer (eBC) patients [2,8] with an acceptable risk–benefit ratio. In premenopausal women with eBC and a low risk of disease recurrence, tamoxifen is the preferred adjuvant ET [2,9]; in those with a high risk, the combination of tamoxifen or an AI with ovarian function suppression (OFS) is recommended [10]; and in postmenopausal eBC patients, therapy with an AI is the treatment of choice [10], since it shows superior results regarding disease-free survival (DFS) [8,11,12,13,14,15] and, in some trials, OS as well [8,13,15].

Since a relevant number of women treated with ET suffer at some point from disease recurrence or progression, novel substances are being investigated to overcome resistance mechanisms by interfering with certain signaling pathways and supporting the efficacy of established therapies [16,17].

This review is designed to give an overview on the recent history of ET, to clarify its role in the present therapy of hormone-receptor-positive HER2-negative BC, and to discuss future directions for further improvement.

## 2. History and the State of the Art of Endocrine Treatment

The beginnings of ET can be dated back to the early 1970s. In the following, the main substances that have been used for decades and still currently build the foundation of any endocrine-based therapy are presented.

### 2.1. Tamoxifen

When the selective estrogen receptor modulator (SERM) tamoxifen first showed positive results in the therapy of hormone-receptor-positive aBC, its development gained more attention [18]. In several randomized trials, tamoxifen demonstrated comparable efficacy with megestrol acetate, which was used to treat BC patients previously. However, tamoxifen presented a better side effect profile, which eventually led to the approval of this therapy in the United States in 1977 [19]. Further analyses showed a reduction in the risk of recurrence when tamoxifen was given as an adjuvant treatment. The optimal therapy duration was subject to several subsequent investigations, which showed a significant trend towards a greater effect with longer treatment, so for many years an adjuvant therapy of five years of tamoxifen was recommended [2].

Later, the ATLAS and aTTom trials demonstrated that an extension in treatment from five to up to ten years leads to a further survival benefit [20,21]; such a treatment duration then became an option, especially for premenopausal patients with a high risk of recurrence [10,22]. In these women, additional ovarian function suppression is also recommended [10,22]. The SOFT and TEXT trials showed improved disease-free survival and overall survival, adding OFS to a treatment with tamoxifen alone [23,24,25].

### 2.2. Aromatase Inhibitors

In the 1970s, investigators also found that estrogen synthesis takes place not only in the ovaries and adrenal glands, but, in postmenopausal women, also in muscle, liver, and adipose tissues by the catalysis of androgens to estrogens through aromatase, an enzyme of the cytochrome P450 family. This knowledge led to the development of the third-generation AIs anastrozole, letrozole, and exemestane to reduce estrogen levels in hormone-receptor-positive BC patients [26]. The comparison between AIs and tamoxifen in postmenopausal women with aBC demonstrated the superiority of AIs and resulted in their approval and establishment in this treatment setting [5,6,7]. Regarding the adjuvant therapy of postmenopausal women suffering from hormone-receptor-positive eBC and the improvement of DFS, AIs’ effect was later analyzed and found again to be superior compared to tamoxifen, resulting in an extension of the approval [8,11,12,13,14,15]. Decades later, in the FATA-GIM3 as well as in the FACE trials, a difference in efficacy between each AI could not be found [27,28].

According to current results, the optimal therapy duration of AIs seems to be at least five years, but due to an ongoing risk of recurrence after the first five years in patients with high-risk BC, a prolongation for up to 10 years in terms of an extended ET seems to further improve DFS [10,29,30]. Nevertheless, statements regarding the exact duration of the extended treatment are partially contradictory, since there are studies that have shown a non-inferiority of seven to eight years of ET compared to 10 years [31] or even no significant difference at all when comparing five versus ten years of AI therapy [32]. An intermittent approach in the SOLE study, however, did not show superior results compared to continuous AI intake [33]. Furthermore, as with tamoxifen, in younger premenopausal women with a high risk of disease recurrence, a combined treatment with an AI and OFS is feasible. This has been shown to improve DFS, but not OS, compared to tamoxifen and OFS as well as to tamoxifen alone [24,25].

### 2.3. Fulvestrant

Another option of ET is fulvestrant, the only approved SERD for the therapy of hormone-receptor-positive aBC in postmenopausal women to date, which joined the ET family in the early years of the millennium. Trials investigating the outcomes of patients that received fulvestrant versus an AI demonstrated that it is at least as effective and safe [34], or even superior [35,36]. This is why it was approved and recommended for the therapy of aBC, especially in the first or second treatment line [34,37,38]. Further analyses included the dosage at which its effect on patients’ outcomes is most favorable, and found that fulvestrant given at 500 mg achieves better results than at 250 mg [34,36]; this then became part of the guidelines [37].

Moreover, ET combination strategies are also of interest. The addition of fulvestrant to an AI, for instance, seems to be associated with an OS benefit, but since it results in a higher incidence of relatively serious adverse events (AEs), this combination treatment did not become the standard of care [39].

## 3. Modern Approaches in Advanced Breast Cancer

**Figure 1:**
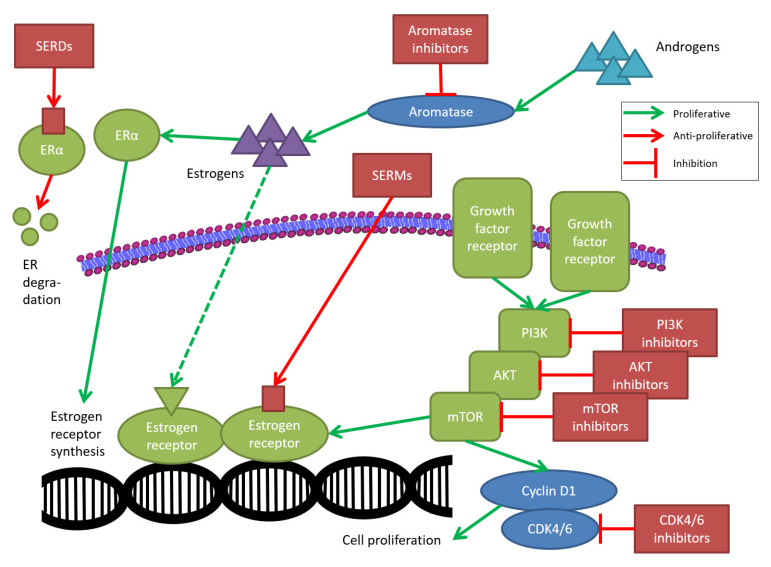
Therapeutic mechanisms of endocrine therapy (simplified representation) [16,17]. AKT: AKT murine thymoma viral oncogene; ER(alpha): estrogen receptor (alpha); CDK4/6: cyclin-dependent kinase 4/6; mTOR: mammalian target of rapamycin; PI3K: phosphatidylinositol 3-kinase; SERD: selective estrogen receptor degrader; SERM: selective estrogen receptor modulator.

Several pathways, resistance mechanisms, and mutations are described in the literature as reasons for disease recurrence or progression. The major pathways include the phosphatidylinositol 3-kinase/AKT murine thymoma viral oncogene/mammalian target of rapamycin (PI3K/AKT/mTOR) signaling pathway, as well as the cyclin-dependent kinase 4/6 (CDK4/6) cell cycle pathway [16,17]. Figure 1 provides an overview of the main cell proliferation mechanisms.

### 3.1. PI3K/AKT/mTOR Signaling Pathway

One of the main substances acting within the PI3K/AKT/mTOR pathway is the mTOR inhibitor everolimus. It was the first agent of the inhibitors of this signaling pathway approved for the therapy of hormone-receptor-positive aBC. Everolimus showed an increase in the time to progression when given in combination with the steroidal AI exemestane [40,41], while a significant improvement in OS could not be demonstrated in the BOLERO-2 trial [42]. The dual mTOR inhibitor vistusertib that, in contrast to everolimus, inhibits not only the mTOR complex 1, but also 2, did not result in a PFS benefit when added to fulvestrant [43]. However, in a small study, sirolimus, also an agent from the mTOR inhibitor family, has so far achieved positive results regarding the PFS, comparable to those under everolimus [44].

Another group of agents inhibiting the above-mentioned signaling pathway that were developed to overcome endocrine resistance are PI3K inhibitors. Buparlisib, such a PI3K inhibitor, was analyzed together with fulvestrant in postmenopausal women with hormone-receptor-positive aBC. At first showing promising results in preliminary studies, its development was later omitted due to a higher rate of grade three and four AEs, seen in the phase III trials BELLE-2 and -3 [45,46,47]. Another PI3K inhibitor that failed to become standard-of-care treatment due to later-phase results is taselisib. Its investigation within the phase III SANDPIPER study led to the conclusion that the combination of taselisib and fulvestrant has no clinical utility given its safety profile and a modest PFS benefit of two months compared to fulvestrant alone [48]. However, in contrast to the above-mentioned substances, with alpelisib a PI3K inhibitor is available for the PIK3CA-mutated, postmenopausal aBC patient, since it shows a statistically significant benefit regarding PFS as well as a meaningful clinical benefit in OS, with an acceptable safety profile at the same time [49,50,51,52]. Its approval is the result of the SOLAR-1 trial, based on which, in current treatment guidelines, the substance is recommended for the therapy of women as well as men with this specific tumor type [53]. Further trials with different treatment combination strategies including CDK4/6 inhibitors, fulvestrant, and alpelisib or novel PI3K inhibitors, such as inavolisib and copanlisib, or even combined PI3K/mTOR inhibitors, such as gedatolisib for instance, will deliver more information about the efficacy and safety of this drug family [54,55,56].

The last group that interferes with this signaling pathway is AKT inhibitors, which is the only group in this pathway that has no approved drug to date. The main substances under current investigation are ipatasertib and capivasertib. Capivasertib is an AKT inhibitor that demonstrated activity in AKT1-mutant hormone-receptor-positive aBC, not only in combination with fulvestrant but also in terms of a monotherapy [57]. PFS in patients dministered capivasertib and fulvestrant was significantly longer than under fulvestrant with a placebo in the phase II trial FAKTION [58]. Therefore, the substance is currently being investigated in the later-phase trial program CAPItello [59]. Ipatasertib, however, failed to prove efficacy regarding PFS when combined with paclitaxel versus paclitaxel alone in the treatment of triple-negative as well as hormone-receptor-positive aBC in the phase III IPATunity130 study [60,61]. Nevertheless, in hormone-sensitive BC patients, research on ipatasertib in combination with fulvestrant and/or a CDK4/6 inhibitor is proceeding [56].

### 3.2. CDK4/6 Signaling Pathway

In 47 human breast cancer and immortalized cell lines representing the known molecular subgroups of breast cancer, palbociclib was tested and showed a differential effect on those cell lines with an emphasize on hormone-receptor-positive and HER2-amplified ones [62]. On the basis of this effect, the further development of CDK4/6 inhibition started [63,64]. In the past few years, the CDK4/6 inhibitors palbociclib, ribociclib, and abemaciclib have been approved and recommended [53] for the treatment of hormone-receptor-positive, HER2-negative aBC on the basis of three large phase III trial programs, PALOMA, MONALEESA, and MONARCH, since all trials show a significant improvement in PFS when combined with an AI or fulvestrant [65,66,67,68,69,70,71] (Table 1). Regarding PFS, CDK4/6 inhibitors in the first and second treatment lines are also superior compared to chemotherapy [72], hence their use increased substantially in the first years after becoming available, while fewer patients started receiving chemotherapy [73,74]. Meanwhile, some of the trials even provided significantly improved OS data, which is an exceptional result in this treatment setting. In the MONARCH-2 trial, for instance, the addition of abemaciclib to fulvestrant significantly prolonged OS in women who had not received chemotherapy and had a maximum of one prior ET for aBC [75]. Under palbociclib and fulvestrant, however, OS was prolonged but did not reach formal statistical significance in the PALOMA-3 trial [76]. There are further results on the efficacy of palbociclib from the PEARL trial, comparing ET with the CDK4/6 inhibitor to chemotherapy. Compared to capecitabine, palbociclib and ET did not demonstrate superiority regarding OS [77]. Besides, in both the study program trials, namely the MONARCH-3 for abemaciclib and PALOMA-2 for palbociclib, any data on OS have not been reported yet and further results are expected. For ribociclib, however, a total of three trials have reported data on OS and show a consistent significant benefit that is independent from its use in the first or second treatment line, pre- or postmenopausal women, or with an AI or fulvestrant as ET partner [78,79,80]. In the MONALEESA-2 study, postmenopausal patients under first-line ribociclib and letrozole achieved a median OS of 63.9 months, the longest OS data reported for aBC to date [78]. Still, the question remains how to treat patients that progress under CDK4/6 inhibition. Novel strategies include, among others, the continuation of CDK4/6 inhibitors through progression as well as triple combinations with PI3K or checkpoint inhibitors [81].

### 3.3. Selective Estrogen Receptor Downregulation

Besides the PI3K/AKT/mTOR and CDK4/6 signaling pathways, the downregulation of estrogen receptor expression also plays a significant role in the treatment of hormone-receptor-positive BC. As mentioned above, fulvestrant is the only approved SERD for the therapy of postmenopausal aBC patients to date. However, since there is a lack of oral bioavailability of fulvestrant, therefore requiring intramuscular injection of it [83,84], other novel SERDs with the potential of oral bioavailability are being investigated [85,86,87,88,89] (Table 2). A first-in-human study on the SERD AZD9496, for instance, showed an acceptable safety profile and a prolongation of disease stabilization in women with hormone-receptor-positive aBC [90], but its preoperative influence on estrogen receptor expression was not superior to fulvestrant within a recent window of an opportunity trial in eBC [91]. Giredestrant, however, has also been analyzed in a similar treatment setting within the neoadjuvant phase II coopERA trial. Compared to anastrozole, after 2 weeks of giredestrant a greater relative reduction in Ki-67 as well as a greater number of tumors achieving complete cell cycle arrest were observed [92]. Results from the phase I/II AMEERA-1 study on the combination of amcenestrant with palbociclib in postmenopausal women with aBC also showed encouraging response and clinical benefit rates [93]. The EMERALD, however, is the first phase III trial with an oral SERD that could show positive results in aBC patients under elacestrant compared to those under standard of care ET regardless of *ESR1* mutations [94].

### 3.4. Germline BRCA1/2 Mutation

Developed on the basis of germline *BRCA1/2* mutations and their role in tumor pathology, poly (ADP-ribose) polymerase (PARP) inhibitors are a further therapy option for patients with advanced HER2-negative disease, including both triple-negative and hormone-receptor-positive BC patients, who make up about 50% of all aBC patients [95]. The OlympiAD trial for olaparib [96] and the EMBRACA trial for talazoparib [97] showed an improvement concerning PFS over a chemotherapy of physicians’ choice. However, in both studies a significant influence on OS was not observed [98,99].

## 4. Adverse Events of and Adherence to Endocrine Treatment

ET is standard in early and advanced therapy settings [10,100,101,102,103,104,105], and therefore the impact of these therapies effects a large number of patients for a long time. The main side effects of clinical interest are musculoskeletal symptoms [106,107], thromboembolic events [108], hot flashes [107], and osteoporosis [109]. While musculoskeletal and vasomotor symptoms usually cease after ET discontinuation, thromboembolic events as well as osteoporosis are side effects with a long-term influence. Additionally, although, in general, ET is one of the best tolerable cancer treatments available, this specific side effect profile often leads to non-compliance [110] and/or non-persistence [111,112,113].

With the advent of combination therapies, new AEs were introduced into ET’s toxicity profile. Under everolimus, for instance, patients may develop stomatitis [41]. In those treated with alpelisib, hyperglycemia and rashes are the most common side effects [52]. Regarding the three CDK4/6 inhibitors, toxicity profiles differ. The rate of grade three/four neutropenia varies depending on the substance, but nevertheless appears in all three CDK4/6 inhibitors. However, there are also substance-specific AEs, such as gastrointestinal toxicities, including diarrhea under abemaciclib, or QTc prolongations under ribociclib [114]. These novel side effect profiles do not seem to affect the QoL in patients with a CDK4/6 inhibitor [115,116,117]; however, they might pose a challenge for treatment management and possibly adherence to these long-term therapies.

Persistence under adjuvant endocrine monotherapy has been investigated in several studies. After one year of letrozole, 13% of eBC patients will already have terminated treatment [118]. Other studies report that almost 50% of women discontinue adjuvant ET with tamoxifen or an AI within the first years of treatment [119]. However, compliance, i.e., whether the intake of the medication is in accordance with a physician’s prescription, and persistence, meaning the intake of the prescribed medication for the recommended duration, both contribute to the patient’s outcome. Studies have shown that patients who do not adhere to the recommended intake and duration of adjuvant ET are more likely to suffer from disease recurrence and have a worse prognosis [111,120]. Regarding terminology, since the definitions of the terms “compliance” and “persistence” vary [111,121,122], this in turn results in difficulties when comparing studies. The term “treatment adherence” is often used in the literature, as in the present study, to cover both [106,111,118].

Several studies have investigated the characteristics of patients who are non-adherent to ET and have tried to determine risk factors, especially in adjuvant therapy [106,111,112,113,118,119,121,123,124,125,126,127], as well as a small number in a metastatic treatment setting [128,129,130].

### 4.1. Endocrine Treatment Adherence in Early Breast Cancer Patients

In an adjuvant situation, age has been found to be a risk factor for non-adherence, with younger and older women in particular being at the highest risk [118,123,126,127]. Regarding body mass index (BMI), there are contradictory results, since some trials show no influence on therapy adherence [111,112], while in the Evaluate-TM study a low BMI was significantly associated with a higher discontinuation rate [118]. Furthermore, comorbidities are shown to negatively influence persistence [118,123,131], but at the same time there are indications for a higher adherence rate in women with diabetes or depression [119]. The prior performance of radiation and chemotherapy, especially when including a taxane, seems to also be associated with women’s ET adherence, although it is not clear to date whether the influence is positive or negative [112,123,124,125]. Regarding tumor characteristics, there are results that suggest tumor stage and size are risk factors for non-adherence [124,126]. Contrary to other trials that did not confirm tumor grade as a risk factor [123,126], one study describes that a higher tumor grade is associated with a better ET persistence [118]. Treatment-associated risk factors mostly include ET side effects, such as musculoskeletal pain syndrome, vasomotor symptoms, or thromboembolic events as reasons for non-adherence [106,107,110,111,112,113]. While patients with musculoskeletal symptoms, mostly due to an AI therapy, are at the highest risk of non-adherence [106,111,112,113], those with pre-existing pain before the beginning of ET seem to be at an additional risk [113], since these women indicate even higher pain values [132].

### 4.2. Endocrine Treatment Adherence in Advanced Breast Cancer Patients

According to the literature, which is scarce, the metastatic situation is similar, although one would expect a higher motivation in patients with a life-threatening disease to continue the recommended treatment. Persistence with palliative ET depends not only on age or the number of comorbidities [128], but also on the presence of ET-induced AEs and previous treatment behavior [129,130,133,134].

## 5. Upcoming Improvements in the Treatment of Early Breast Cancer

The above-mentioned resistance mechanisms play not only a role in the advanced therapy setting, but also for neo-/adjuvantly treated eBC patients. Substances already approved for aBC are now subject to eBC trials.

### 5.1. CDK4/6 Inhibitors as a Possible New Cornerstone

CDK4/6 inhibitors have been investigated in high- and intermediate-risk eBC patients in studies of one to three years in combination with neo-/adjuvant ET with the aim to enhance the DFS rate (Table 3). However, the addition of one year of palbociclib to adjuvant ET in women with residual disease after neoadjuvant chemotherapy, as in the PenelopeB trial, did not improve DFS [135]. Neither did two years of palbociclib in patients with stage II–III disease in the PALLAS trial [136]. Two years of abemaciclib, in contrast, delivered in the primary outcome analysis of the monarchE trial, where women with node-positive eBC and a high-risk profile received the CDK4/6 inhibitor in addition to ET, positive results. While under ET alone after a median follow-up of 19 months, 89.3% of eBC patients were cancer-free, under abemaciclib it was a total of 92.3% (HR 0.71; 95% CI 0.58–0.87; and *p* = 0.0009) [137]. With 27 months of median follow-up the invasive DFS benefit was maintained (HR 0.70; 95% CI 0.59–0.82; and *p* < 0.0001) [138]. On this basis abemaciclib has been approved by the FDA for the treatment of patients with hormone-receptor-positive, HER2-negative, node-positive eBC at a high risk of recurrence and a Ki-67 score of ≥20%. Results from the NATALEE trial with adjuvant ribociclib for a total of three years in high- and intermediate-risk eBC patients [139] are expected in the near future, which will help investigate whether the significant OS benefit seen in the MONALEESA-2/-3/-7 trials for aBC is transferable to the early treatment setting.

### 5.2. CDK4/6 Inhibitors Instead of Neo-/Adjuvant Chemotherapy

Even more interesting than the adjuvant use of CDK4/6 inhibitors together with ET is the vision of this combination replacing neo-/adjuvant chemotherapy. The phase II CORALLEEN study compared neoadjuvant ET with six cycles of letrozole and ribociclib to four cycles of a chemotherapy regime containing anthracycline and taxane. At the time of surgery, many of the tumors initially classified as luminal B were then measured to be luminal A tumors [140]. Neoadjuvant abemaciclib within the phase II neoMONARCH trial also demonstrated a certain potential of cell cycle arrest and immune activation [141], as did palbociclib in the neoadjuvant phase II trials neoPAL [142] and neoPalAna [143]. Larger phase II trials, such as CARABELA with abemaciclib or Appalaches with palbociclib [56], and the phase III study ADAPTcycle with ribociclib [144] will yield more information about the comparability of chemotherapy with a CDK4/6-inhibitor-based treatment with regard to efficacy and safety.

### 5.3. Further Approaches

Since the present review focuses on hormone-receptor-positive, HER2-negative BC, Table 3 provides an overview of the key ongoing trials that include a CDK4/6 inhibitor in the adjuvant treatment of this specific tumor type. However, CDK4/6 inhibitors are also being investigated in several combinational regimes for HER2-positive, HER2-low, or HER2-enriched [145] disease, but this is not the subject of this manuscript.

The role of PI3K inhibitors in eBC remains unclear. LORELEI, a phase II trial, was initiated to investigate the efficacy of taselisib combined with letrozole in the neoadjuvant ET setting. An objective response due to the addition of this PI3K inhibitor was demonstrated, but it was not sufficient enough to be recommended [146]. Alpelisib, in contrast to its efficacy in aBC, did not show any benefit when added to neoadjuvant ET in the NEO-ORB trial [147]. The development of the newer PI3K inhibitors inavolisib, copanlisib, and gedatolisib will reveal more about their potential significance for eBC.

Oral SERDs are also not only the subject of aBC trials but are analyzed in patients with eBC as well (Table 2). The phase III AMEERA-6 study, for instance, evaluates amcenestrant versus tamoxifen for postmenopausal women, with hormone-receptor-positive eBC unable to continue adjuvant AI therapy [148]. Giredestrant is being compared to physicians’ choices of ET in women with hormone-receptor-positive, HER2-negative eBC within the phase III lidERA trial [149]. These trials will show whether oral SERDs can become established substances of adjuvant ET.

Moreover, in *BRCA*-mutated patients with hormone-receptor-positive disease, there is also evidence that PARP inhibitors may enhance ET efficacy through a synergistic effect, since *BRCA* mutations have been linked to transcriptional function of hormone receptors [150,151]. The OlympiA trial is the first to show a benefit in DFS in women with HER2-negative eBC and a *BRCA1/2* mutation receiving one year of adjuvant olaparib. The 3-year DFS of the total population was 85.9% in the olaparib group and 77.1% in the placebo group (HR 0.58; 99.5% CI 0.41–0.82; and *p* < 0.001). However, a significant difference in OS rates could not be demonstrated. Looking only at hormone-receptor-positive, HER2-negative eBC patients who received olaparib for one year in addition to ET, compared to those included after adjuvant chemotherapy on the basis of node-positivity (HR 1.36; 95% CI 0.41–4.71), the subgroup of women who were enrolled due to residual disease after neoadjuvant chemotherapy benefitted the most (HR 0.52; 95% CI 0.25–1.04) [152]. Particularly in this patient population, combinational treatments might therefore be of interest in the future.

However, to be effective, each therapy requires patients’ adherence, especially in view of the cost–benefit ratio of novel substances and known unsatisfying adherence rates of standard ET. Therefore, to ensure women’s compliance and persistence and to improve their outcome with the help of expensive combinational regimens, the necessity of patients’ understanding of their recurrence risk, compliance programs, and digital health solutions, continuous treatment and side effect monitoring as well as a healthy patient–physician relationship will gain in importance with each novel therapy.

## 6. Conclusions

ET of BC, in both an early and advanced setting, has existed for several decades, and has consisted of the basic substances tamoxifen, an AI, or fulvestrant. Prolonging overall survival in aBC and now also entering the eBC treatment setting, CDK4/6 inhibitors will be the first novel substances to revolutionize this therapy area over the coming decades. Despite ET adherence rates being in need of improvement, in general it is one of the best tolerable cancer therapies available. Continuous investigation serves to improve patients’ outcomes and modernizes the current standard of care. Considering several recent successes in treatment efficacy, the rapid development of new drugs in the past few years, and their prompt implementation in treatment guidelines, the continuous improvement of ET is already a reality.

## Figures and Tables

**Table 1:** Efficacy of CDK4/6 inhibitors in hormone-receptor-positive, HER2-negative aBC phase III trials (in alphabetical and numerical order).

Treatment Combination	Study Name	Sample Size	Randomization	Median PFS in Months					Median OS in Months				
				With CDK4/6 Inhibitor	Without CDK4/6 Inhibitor	HR	95% CI	Statistically Significant as per Protocol	With CDK4/6 Inhibitor	Without CDK4/6 Inhibitor	HR	95% CI	Statistically Significant as per Protocol
**ET +/− abemaciclib**	MONARCH-2 [68,75]	669	2:1	16.4	9.3	0.55	0.45–0.68	Yes	46.7	37.3	0.76	0.61–0.95	Yes
	MONARCH-3 [69]	493	2:1	28.2	14.8	0.54	0.42–0.70	Yes	Not reported yet				
**ET +/− dalpiciclib**	DAWNA-1 [82]	361	2:1	13.6 ^1^	7.7 ^1^	0.45 ^1^	0.32–0.64 ^1^	Yes ^1^	Not reported yet				
**ET +/− palbociclib**	PALOMA-2 [66]	666	2:1	24.8	14.5	0.58	0.46–0.72	Yes	Not reported yet				
	PALOMA-3 [67,76]	521	2:1	9.5	4.6	0.46	0.36–0.59	Yes	34.9	28.0	0.81	0.64–1.03	No
**ET +/− ribociclib**	MONALEESA-2 [71,78]	668	1:1	25.3	16.0	0.57	0.46–0.70	Yes	63.9	51.4	0.76	0.63–0.93	Yes
	MONALEESA-3 [65,79]	726	2:1	20.5	12.8	0.59	0.48–0.73	Yes	53.7	41.5	0.73	0.59–0.90	Yes
	MONALEESA-7 [70,80]	672	1:1	23.8	13.0	0.55	0.44–0.69	Yes	58.7	48.0	0.76	0.61–0.96	Yes

HER2: human epidermal growth factor receptor 2; aBC: advanced breast cancer; CI: confidence interval; ET: endocrine treatment; HR: hazard ratio; OS: overall survival; and PFS: progression-free survival. ^1^ As assessed by an independent review committee.

**Table 2:** Selection of novel SERDs under clinical development in phase III eBC/aBC trials (in alphabetical order; based on clinicaltrials.gov, accessed on 22 September 2021) [56].

Substance Name	Original Substance Code	Trial Program
Amcenestrant [85]	SAR439859	AMEERA
Camizestrant [88]	AZD9833	SERENA
Elacestrant [87]	RAD1901	EMERALD
Giredestrant [86]	GDC-9545	persevERA (aBC) and lidERA (eBC)
- ^1^	LY3484356 [89]	EMBER

SERD: selective estrogen receptor degrader; eBC: early breast cancer; and aBC: advanced breast cancer. ^1^ Data unknown at the time of writing this manuscript.

**Table 3:** Selection of key ongoing CDK4/6 inhibitor trials on hormone-receptor-positive, HER2-negative eBC (in alphabetical order; based on clinicaltrials.gov, accessed on 22 September 2021) [56].

CDK4/6 Inhibitor	Abemaciclib				Dalpiciclib	Palbociclib ^1^			Ribociclib		
**Study name**	ADAPTlate	CARABELA	monarchE	POETIC-A	SHR6390-III-303	Appalaches	POLAR	TRAK-ER	ADAPTcycle	LEADER	NATALEE
**Study code**	NCT04565054	NCT04293393	NCT03155997	NCT04584853	NCT04842617	NCT03609047	NCT03820830	NCT04985266	NCT04055493	NCT03285412	NCT03701334
**Phase**	III	II	III	III	III	II	III	II	III	II	III
**Brief study summary**	Adjuvant abemaciclib + SOC ET vs. SOC ET	Neoadjuvant abemaciclib + SOC ET vs. chemotherapy	Adjuvant abemaciclib + SOC ET vs. SOC ET	Adjuvant abemaciclib + SOC ET vs. SOC ET	Adjuvant dalpiciclib + SOC ET vs. SOC ET	Adjuvant palbociclib + SOC ET vs. chemotherapy followed by SOC ET	Adjuvant palbociclib + SOC ET vs. SOC ET	Adjuvant palbociclib + fulvestrant vs. SOC ET	Adjuvant ribociclib + SOC ET vs. chemotherapy followed by SOC ET	Adjuvant intermittent ribociclib + SOC ET vs. continuous ribociclib + SOC ET	Adjuvant ribociclib + SOC ET vs. SOC ET
**Main patient population criteria**	High risk	High and intermediate risk	High risk	High risk	High risk	High risk	Local/regional recurrence	High risk	Intermediate risk	High and intermediate risk	High and intermediate risk
	Pre- and postmenopausal	Pre- and postmenopausal	Pre- and postmenopausal	Postmenopausal	Node-positive	≥70 years old	Pre- and postmenopausal	Pre- and postmenopausal	Pre- and postmenopausal	MRD based on ctDNA	Pre- and postmenopausal
	Female only	Female only	Male patients allowed	Female only	Pre- and postmenopausal	Male patients allowed	Male patients allowed	Male patients allowed	Female only	Pre- and postmenopausal	Male patients allowed
					Female only						
**Randomization**	1:1	1:1	1:1	1:1	1:1	2:1	1:1	1:1	3:2	1:1	1:1
**Duration of CDK4/6i intake**	2 years	1 year	2 years	2 years	- ^2^	2 years	3 years	2 years	2 years	1 year	3 years
**Number of patients**	1250	200	5637	2500	4350	366	400	1100	1670	231	5101
**Primary endpoint**	iDFS	RCB	iDFS	iDFS	iDFS	D-RFI	iDFS	ctDNA, iDFS	iDFS	Safety	iDFS

HER2: human epidermal growth factor receptor 2; CDK4/6i: CDK4/6 inhibitor; ctDNA: circulating tumor DNA; eBC: early breast cancer; D-RFI: distant recurrence-free interval; ET: endocrine treatment; iDFS: invasive disease-free survival; MRD: minimal residual disease; RCB: residual cancer burden; and SOC: standard of care. ^1^ Further trials with palbociclib in eBC, such as PenelopeB [135] and PALLAS [136], are not listed here due to already reported negative results. ^2^ Data unknown at the time of writing this manuscript.
